# Northern and southern blacklegged (deer) ticks are genetically distinct with different histories and Lyme spirochete infection rates

**DOI:** 10.1038/s41598-020-67259-0

**Published:** 2020-06-24

**Authors:** Guang Xu, Ben Wielstra, Stephen M. Rich

**Affiliations:** 1 Department of Microbiology, University of Massachusetts, Amherst, United States of America; 2 Institute of Biology Leiden, Leiden University, Leiden, The Netherlands; 3 Naturalis Biodiversity Center, Leiden, The Netherlands

**Keywords:** Microbiology, Molecular biology, Zoology

## Abstract

Lyme borreliosis (LB) is the archetypal emerging zoonosis and is dependent on transmission by ticks in the genus *Ixodes*. Understanding the origin, maintenance, and spread of these ticks contributes much to our understanding of the spread of LB and other disease agents borne by these ticks. We collected 1232 *Ixodes scapularis* ticks from 17 east coast sites ranging from New Hampshire to Florida and used mtDNA, three nuclear genetic loci, and incorporated Bayesian analyses to resolve geographically distinct tick populations and compare their demographic histories. A sparse, stable, and genetically diverse population of ticks in the Southeastern US, that is rarely infected with the agent of LB is genetically distinct from an abundant, expanding, and comparatively uniform population in the Northeast, where epidemic LB now constitutes the most important vector borne disease in the United States. The contrasting geography and demography of tick populations, interpreted in the context of the geological history of the region, suggests that during the last glacial period such ticks occupied distinct refugia, with only the northern-most site of refuge giving rise to those ticks and pathogens now fueling the epidemic.

## Introduction

Lyme disease is the most commonly reported vector borne disease in North America though its risk varies widely by geographic region^1^. *Ixodes scapularis* is the main vector tick of *Borrelia burgdorferi*, the bacterium that causes Lyme disease. In the United States, most human Lyme disease cases are reported in the Northeast and upper Midwest, while cases in western and southern regions of the country are low^2^. This contrast is also evident in the entomological record where the frequency of *B. burgdorferi* is 2–8% in the main vector *I. scapularis* adult ticks in the South^3,4^, compared to infection rates averaging 50% in New England^5^. Environmental factors may affect survival rates of immature *I. scapularis* and the geographical distribution of Lyme disease^6^. In addition to the persistent difference in Lyme infection rates, there are major differences of questing behavior, human attacking rate, host preferences, and survival rate between northern and southern populations of *I. scapularis* ticks^7–11^. There are also vast differences in abundance, with *I. scapularis* tick densities in the South being much lower than in the North^4,12^.

Due to these phenotypic variations, it is not surprising that large genetic differences occur between north and south *I. scapularis* populations. In 1985, the original description of the north population as a new species *I. dammini* was driven in part by the report of an abundant *I. ricinus*-like tick with northern distribution several hundred miles disjunct from that of the southern *I. scapularis*^13^. However, the morphological differences between northern and southern populations were cryptic, tending to manifest only during particular life stages. In 1993, Oliver and colleagues suggested that *I. dammini* should be reduced to a junior synonym of *I. scapularis*^14^. Population genetic studies showed no differences in chromosome morphology or allozyme frequencies among populations^14,15^. The nuclear ribosomal DNA sequences of the ITS-l and ITS-2 regions were also continuously distributed among populations^16,17^. However, the distribution of mitochondrial haplotypes is distinct and forms two clades: (1) the American clade, found in the northeastern US down to the Carolinas, and (2) the Southern clade, which overlaps in the Carolinas and extends into the southeastern states. More genetic variation and diversity have been observed in ticks collected from southern states^18–21^ and extremely abundant single nucleotide polymorphisms (SNPs) have been found in *I. scapularis*^22^. SNPs analysis for ten ticks showed that samples from New Jersey and Virginia formed a homogeneous group with low genetic diversity, whereas southern ticks from Georgia and Mississippi consisted of two separate groups, each with high genetic diversity. SNPs analysis also revealed a predominantly North to South gene flow^23^.

While the population genetic structure of blacklegged ticks is a fundamental basis for understanding and predicting Lyme disease epidemiology, relevant studies have mainly utilized phylogenetic methods based on mitochondrial markers from a small number of individuals at limited collection sites. As a result, we still do not know the border between the northern and southern populations. Studies of nuclear, segregating genetic markers that could elucidate the evidence for genetic exchange across the broad geographic range of the east coast of the United States are limited^24^. Although ticks from northern and southern populations appear to be equally competent *B. burgdorferi* vectors in laboratory conditions^25,26^, data on the prevalence of the Lyme spirochete has not been linked to mitochondrial Southern clade ticks. The present study is based on mtDNA and three nuclear genetic loci and incorporated Bayesian analyses to detect evidence of population structure and geographically distinct tick populations. We have also used ecological niche modeling to determine the historical distribution and spread of these ticks. We found that northeastern (northern) and southeastern (southern) *I. scapularis* tick populations are genetically distinct with different histories and Lyme spirochete infection rates.

## Results

### Species and PCR amplification

**Figure 1:**
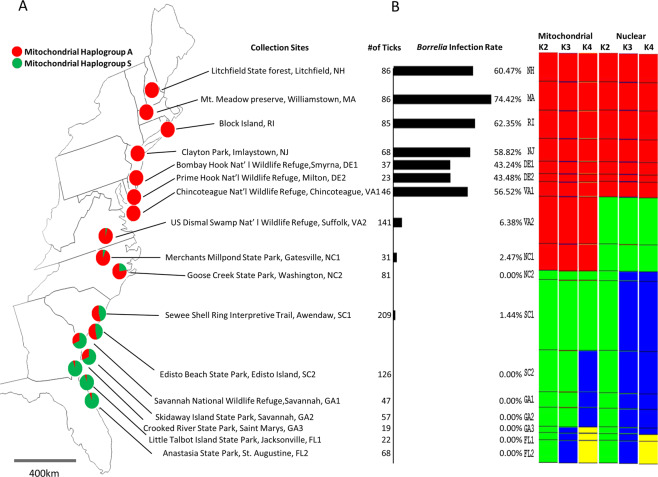
Collection sites, percentage of mitochondrial Haplogroups, *Borrelia* infection rates and BAPS population structures. (**A**) Colored circles are 17 collection sites of 1232 ticks from eastern US. Red and green colors show the percentage of mitochondrial Haplogroup A and S. (**B**) Total tick numbers in each collection site with *Borrelia* infection rate. Population structure analysis is based on mitochondrial and 3 nuclear genes using BAPS. Each color represents one population.

A total of 1236 adult ticks were flagged from vegetation from 17 east coast sites in November to December 2007 (Fig. 1). Four ticks collected at Awendaw, South Carolina were not identified as the species of interest (*I. affinis* N = 3; and *I. brunneus* N = 1) based on the mitochondrial 16 S sequences. Fragments of mitochondrial 16 S ribosomal RNA (mt DNA 16 S), nuclear (nuc) Calreticulin (CRT), Glycerol-3-phosphate dehydrogenase (G3PD) and Isocitrate dehydrogenase (IDH) genes were amplified for 1232 adult *I. scapularis* ticks.

### Phylogeny and distribution of haplotypes

**Figure 2:**
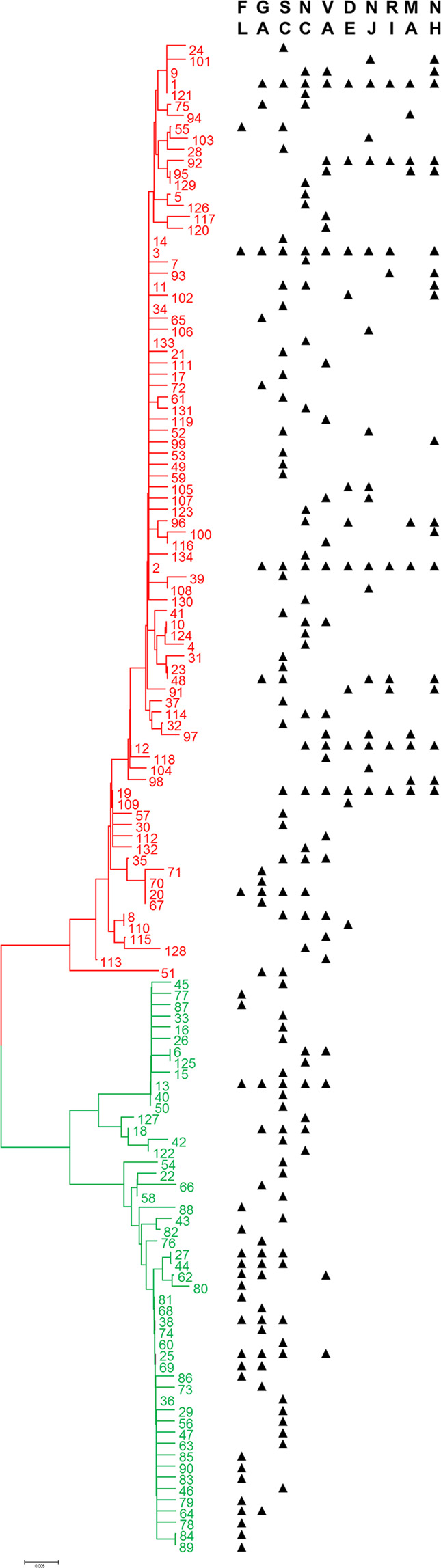
Phylogenetic tree of mtDNA haplotypes. The red color represents Haplogroup A and the green color Haplogroup S. Triangles show geographic distribution of each haplotype (Software used to generate the maximum likelihood (ML) tree was PAUP Version 4.0b10, https://paup.phylosolutions.com).

In agreement with the findings of previous studies^18,19^, the phylogenetic analysis of 16 S gene revealed two mitochondrial haplogroups (Fig. 2). The proportion and geographic distribution of these two haplogroups clearly shows that one is distributed mainly in the North, but extending to the South, while the second is restricted only to the Southeast locations. From Suffolk, Virginia to Florida the haplotype ratios appear as a latitudinal cline, with the frequency of the southern haplogroup starting at 94.1% in St. Augustine, Florida and declining to 3.5% at Suffolk Virginia, and no individuals within that haplogroup occurring further north (Fig. 1).

Analysis of mitochondrial 16 S sequences yielded 134 unique haplotypes. The greater number of haplotypes was detected in the southern locations (Fig. 2) (Supplementary S2 Table 1). The cluster analysis of mitochondrial haplotypes showed two groups. The samples from Florida, Georgia and South Carolina formed one group, and the samples from North Carolina, Virginia, Delaware, New Jersey, Rhode Island, Massachusetts and New Hampshire formed another group. Clearly, the 16 S haplotype distributions are different between the South and the North (Fig. 2).

**Figure 3:**
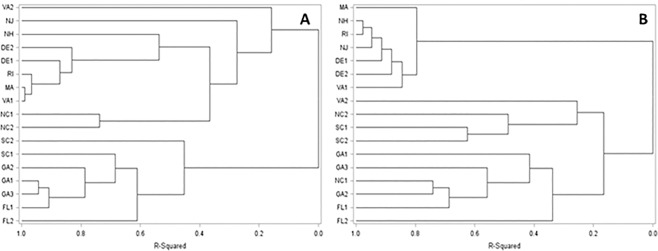
Cluster Analysis of mtDNA (**A**) and nucDNA (**B**) haplotypes distribution among 17 east coast collection sites (New Hampshire: NH; Massachusetts: MA; Rhode Island: RI; New Jersey: NJ; Delaware: DE1 and DE2; Virginia: VA1 and VA2; North Carolina: NC1 and NC2; South Carolina: SC1 and SC2; Georgia: GA1, GA2 and GA3; Florida: FL2 and FL2), (cluster analysis was performed by SAS software Version 9.0, https://www.sas.com).

DNA sequences of 3 nuclear genes yielded 342, 201 and 124 unique haplotypes for CRT, IDH and G3PD, respectively. G3PD, CRT and IDH haplotype distributions also showed significant differences between the South and the North (Fig. 3). However, compared to the mitochondrial gene haplotype distribution, the cluster analysis of nuclear gene haplotype distribution suggested the boundary between southern and northern populations to be positioned more to the north, allocating the two Virginia populations to the southern population.

### Population comparison

The mitochondrial 16 S gene, and nuclear genes CRT and IDH supported a separation between northern and southern populations when pairwise differences were used to analyze the population relationships (See Supplementary S1 Fig. A to D). The evaluated Fst values indicated limited gene flow between the southern and the northern populations. However, pairwise comparisons of Fst and Nei’s distance of G3PD gene showed no significant correlations between geographic locations and pairwise differentiation. Strong population structure was detected using AMOVA among populations, both within and between the North and South (Supplementary S2 Table 2).

### Demographic history

According to the mismatch distribution analysis of 4 individual genes, the spatial expansion model could not be rejected for all 17 populations. However, the demographic model showed different results (Supplementary S2 Table 3). A mismatch distribution is the frequency distribution of the observed number of differences between pairs of haplotypes. The mitochondrial 16S gene suggested a sudden expansion for northern populations and stable tick populations in Florida, Georgia and South Carolina and North Carolina. The mismatch distribution of CRT and IDH sequences also indicated a different demographic history between southern and northern populations.

### Bayesian analysis of population structure

The seventeen populations were clustered using Structure and BAPS software population mixture analysis. Both Structure and BAPS software suggest K = 4 for nuclear genes and K = 2 for mtDNA gene. The mitochondrial and combined nuclear data all suggested two major blacklegged tick population clusters along the eastern U.S. coast, with a single population containing NH, MA, RI, NJ, DE1, DE2 and VA1 and 2–3 possible subpopulations in the Southern area (Fig. 1). The mitochondrial and nuclear genes identified different boundaries of these two populations. Ticks (VA2) from Suffolk, Virginia belonged to the northern population based on mtDNA but to the southern group based on nuclear DNA.

### Borrelia infection rate

The *Borrelia* infection rate ranged from 43.24 to 74.42% in NH, MA, RI, NJ, DE1, DE2 and VA1 tick populations (Fig. 1). The highest infection rate was 74.42% in Massachusetts. The infection rates of the two Virginia populations that belong to the southern population based on nuclear DNA but to the northern population based on mtDNA were very different, with an infection rate of 56.52% in Chincoteague (VA1) population and 6.38% in the Suffolk (VA2) population. In populations allocated to the southern group based on both mtDNA and nuclear DNA the infection rate was very low, ranging from 0 to 2.47%. The *Borrelia* infection was strictly limited to individuals with mitochondrial Haplogroup A; no Haplogroup S individuals were found positive for *Borrelia*.

### Ecological niche model

**Figure 4:**
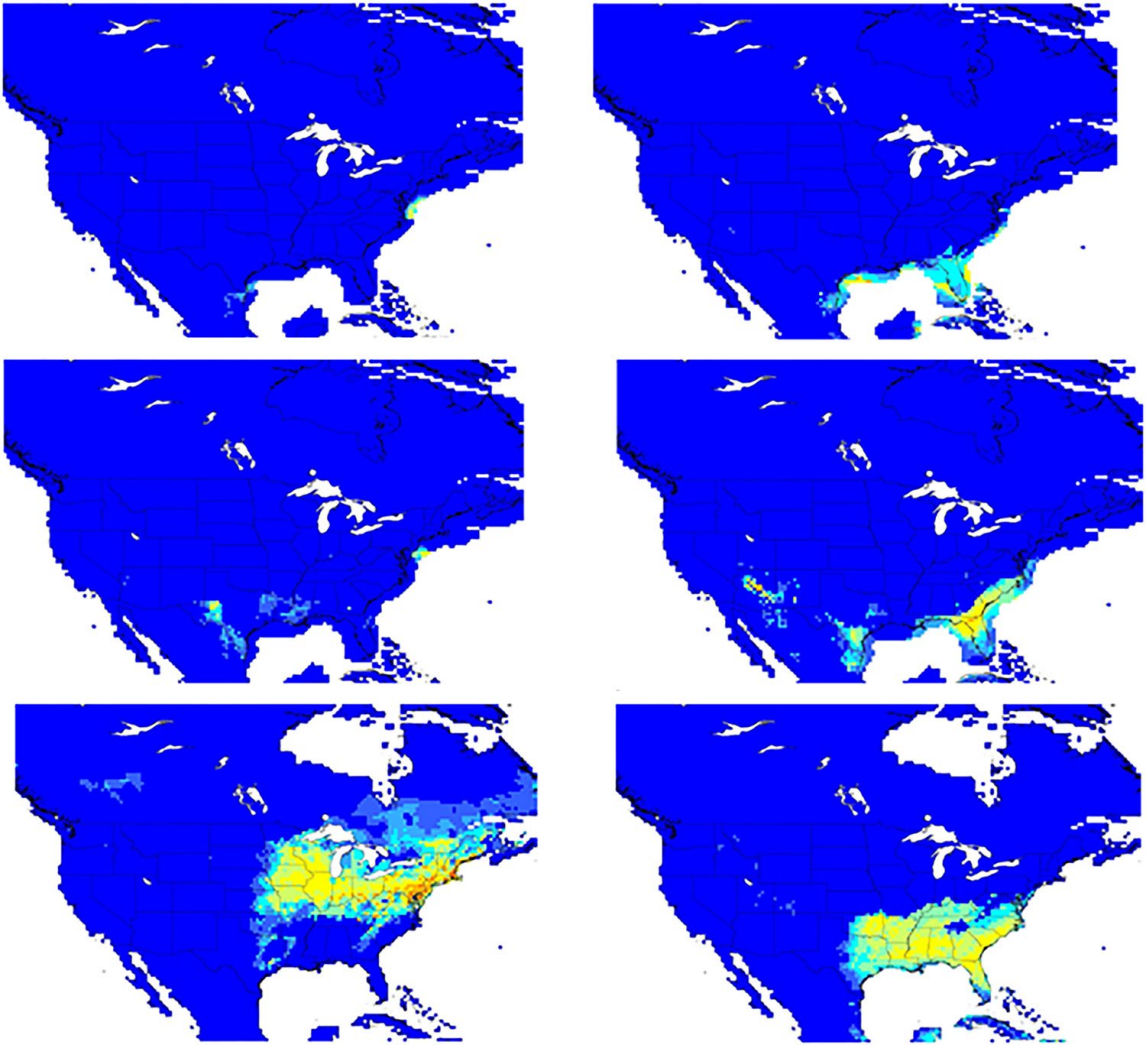
The predicted distribution for  the northern (left) and southern population (right) of *Ixodes scapularis* at the Last Glacial Maximum (top), the mid-Holocene (middle) and the present day (bottom), based on ecological niche modeling. The warmer a grid cell’s color, the higher its predicted suitability (Software used to draw the map was Maxent Version 3.3.3k, https://biodiversityinformatics.amnh.org/open_source/maxent/).

Projection of the ecological niche models (AUC values for the northern and southern population were 0.94 and 0.96) on the two Last Glacial Maximum climate reconstructions (CCSM and MIROC) suggested that the northern tick population was derived from a small refuge located just south of the Pleistocene ice sheet on the Atlantic continental shelf off the current Delmarva Peninsula. The southern *I. scapularis* population probably originated from a suitable refuge located in the southern coastal plain of today’s Florida, Georgia and South Carolina (Fig. 4).

## Discussion

Based on haplotype distribution, population comparison, phylogenetic analysis, demographic history and Bayesian analysis of population structure, we conclude that there are two distinct blacklegged tick populations (northern and southern) along the US eastern coast, with distinct *Borrelia* infection rates and post-glacial histories.

In agreement with previous mtDNA studies^18–20^, this study not only suggested that blacklegged ticks are geographically structured along the east US coast, but also suggested that two distinct clades meet at North Carolina and Virginia. The southern *I. scapularis* population is characterized by greater heterogeneity and absence of widely distributed haplotypes. In contrast to the results from the ITS-2 gene^17^, analysis of three nuclear genes confirms the existence of two major structured populations of *I. scapularis* ticks. Both mitochondrial and nuclear genes are comparatively homogeneous in northern *I. scapularis* populations, but more diverse in southern *I. scapularis* populations. We found nuclear genes suggested a boundary between the northern and southern population in Virginia, whereas mtDNA suggested this boundary to be positioned in North Carolina.

The demographic model strongly supports a recent and rapid increase in population size for all Northeastern sites. By contrast, only several southern sites (Florida, Georgia and South Carolina) showed no evidence of changes in historical population size. The ecological niche model suggests that there were two possible distinct refugia in eastern North America during the Last Glacial Maximum. The northern *I. scapularis* populations are suggested to be derived from a small refugia located to the south of the Pleistocene ice sheet and on the Atlantic continental shelf off the current Delmarva Peninsula. Although this region has been submerged as sea level raised from melted glacial ice, it was a broad coastal plain and well covered with land vegetation during the Last Glacial Maximum^27^. The southern *I. scapularis* population may have originated from a suitable refuge that is more to the South, located in the Southern coastal plain. The later expansion of the southern population seemed to occur through much of the Southeastern quadrant of the United States. However, since *I. scapularis* is expanding its range and has recently shown tolerance for colder conditions in northern US and southern Canada, the current distribution of *I. scapularis* may not represent the full spectrum of its habitable environment, and the inferred historical range might be underestimated. The New York-Connecticut region^20^ and the areas further north of Delmarva have been suggested as a likely location of refugia populations of *I. scapularis*^28^.

We detected a homogenous northern population and a heterogeneous southern population. The heterogeneity of the southern population is due to introgression from the northern population. In agreement with previous studies^18,19^, we found that the two major mitochondrial haplogroups co-occur throughout the south. All northern ticks carry only Haplogroup A, while Haplogroups A and S occur mixed throughout the South. This has previously been explained as evidence of continuous gene flow among populations and a northward movement of the southern population^3^. We, however, found that the relative frequency of A and S haplogroups is a latitudinal cline with S increasing towards the South. The introgression of the northern population mitochondrial DNA might suggest that gene flow between two tick populations has migrated southwards. Positive selection or migrating birds may play an important role for the directional gene flow because they would carry ticks southward more effectively than northward^28^. However, the expansion of *I. scapularis* includes southward as well as northward expansion. For example, the recent northward invasion of *I. scapularis* and *B. burgdorferi* in Canada has occurred via the introduction by migratory birds^29^. The environmental conditions and selective pressure from warmer temperatures may change the host-seeking behavior of southern tick population^6^. Alternatively, the northern mtDNA in the Southeastern population may represent a genetic footprint left after the southern population displaced an expanding northern population^30,31^. Bayesian Analysis of Population Structure revealed two populations with a possible hybridization zone located in Virginia.

We found that the southern *I. scapularis* populations appear to be at a demographic equilibrium, while northern populations have gone through a recent demographic expansion, especially after glacial retreat. However, human activities have accelerated the expanding of *I. scapularis* ticks in the eastern United States and southern Canada during the 20th century^32^. *I. scapularis* was first collected on Naushon Island, Massachusetts in 1920. With the return of white-tailed deer to other areas of the Northeast and Midwest, the range of *I. scapularis* began expanding^28^. During the 1960s, focal *I. scapularis* infestations were recognized on Long Island and Nantucket Island in the Northeast and in northwestern Wisconsin. By the mid-1970s, the known range of *I. scapularis* had expanded, particularly extending in a southerly direction along the middle-Atlantic coast^28^. Within 100 years, this tick has largely spread throughout most of the eastern United States and into southern Canada^32^. The expansion of *I. scapularis* and *B. burgdorferi* is determined by distinct environmental factors: climate, habitat and host range and abundance^29,33,34^. In this study, only climatic factors were included in the ecological niche models.

The most interesting finding is that a high infection rate of *Borrelia* (43–74%) is mostly linked to the northern *I. scapularis* population and where *Borrelia* does occur in the southern *I. scapularis* population (as defined by nuclear DNA), it is always found linked to the northern mtDNA type. The infection rate drops substantially to 0–6% with a change of the nuclear DNA defined population boundary in Virginia. Whether the differences in the infection rate is environmentally determined, is a heritable trait for two populations or is a combination of genetic traits and hosts needs further study. For example, warmer temperatures and a lower density of host population may change the host-seeking behavior of southern tick population and eventually lower the incidence of Lyme disease in areas at low altitudes^6^. However, environmental factors alone cannot explain the virtual absence of infection rate in *I. scapularis*. Although different hosts were believed to be the reason for low infection rates in the South, a comparative study between two sites in eastern Virginia^35^ showed the infection of white-footed mice is 66.5% in the coastal site and 15.5% in the inland sites. *Borrelia* infection is 35.7% and 0% in the nymphal ticks removed from these mice in the coastal site and in the inland sites, respectively. Recently, the absence of detectable *B. burgdorferi* infection was found among blacklegged ticks in North Carolina^36^ and Tennessee^37^. These may suggest a genetic basis of tick behaviors that influence the epidemiology of vector-borne pathogens. It is very likely that the Northeastern tick population is the principal tick population responsible for transmitting Lyme disease. This same population is solely carrying two zoonotic agents in the Eastern US: *Anaplasma phagocytophilum* and *Babesia microti*^13^.

In this paper, we only sampled adult ticks from eastern coastal sites. Tick-borne disease in the Midwest of the US and Canada is increasing as *I. scapularis* populations continue to spread and grow^29,38^. It is worth noting that tick population ecology and spirochete transmission patterns are different between the northeast and northern Midwest. The two foci of *B. burgdorferi* in the northeastern and midwestern have a shared past and once belonged to an admixed population but are now isolated^11,39,40^. Transport of *B. burgdorferi* between northeastern and midwestern *I. scapularis* populations appears to be relatively infrequent.

The suggestion of a genetic basis to the transmission of *Borrelia* by Northeastern populations of *I. scapularis* is of significance for public health. We believe that, currently, the risk of acquiring Lyme disease through *I. scapularis* population is low in the South. First because they are low in density and they rarely bite humans^7–9^; more importantly, this population is not likely to be carrying the Lyme spirochete. However, this does not mean that there are no enzootic cycles of spirochetes in the South. Enzootic transmission of Lyme disease spirochetes among rodents and ticks has been documented in Southern and South-central states. Although they rarely bite humans, the sympatric tick species *I. affinis*, *I. minor* and *I. dentatus* may maintain a different enzootic cycle in the South^41^. In this study, two of three collected *I. affinis* ticks are *Borrelia* positive. In North Carolina, *Borrelia* DNA was detected in 63.2% of 155 *I. affinis*, but in 0% of 298 *I. scapularis*^36^; so-called “*Ixodes scapularis*” with a 35% *Borrelia* positive rate was confirmed as a non-accurate identification of *I. affinis*^42^. The current and potentially enhanced future role of the southern blacklegged tick population *I. scapularis* in Lyme epidemiology requires additional studies. Monitoring dynamics of the two populations will help us to predict future changes of tick and tick-borne pathogens.

## Materials and Methods

### Sampling natural tick populations

Adult ticks were collected from 17 sites in 10 states (New Hampshire: NH; Massachusetts: MA; Rhode Island: RI; New Jersey: NJ; Delaware: DE1 and DE2; Virginia: VA1 and VA2; North Carolina: NC1 and NC2; South Carolina: SC1 and SC2; Georgia: GA1, GA2 and GA3; Florida: FL2 and FL2) throughout the eastern United States by dragging a 1-m^2^ white flannel flag through vegetation in November - December 2007 (Fig. 1). Ticks were kept alive until we returned to the laboratory, then frozen individually at −80 °C. Total DNA was extracted from individual ticks using the Epicenter Master Complete DNA & RNA Purification Kits (Epicenter Technologies, Madison, WI), following the manufacturer protocols.

### Gene markers

We examined multiple genetic loci, including partial sequences of one mitochondrial gene and three nuclear genes. Both mitochondrial and nuclear gene primers were developed from the available sequences in GenBank. Primers TK16S_105F (5′-CGGTCTGAACTCAGATCATGTA-3′) and TK16S_546RC (5′-AATTGCTGTGGTATTTTGACTATAC-3′) were used to amplify a mitochondrial 16 S (441 bp) region. PCR was performed at 95 C 15 sec, 45 C 15 sec and 72 C 60 sec for 40 cycles. Primers CRT_849F (5′-TGGACGAGCCGATGGGTA-3′) and CRT_1657RC (5′-GGGCTGTACTCTGGGTTATCGA-3′), G3PD_3574F (5′-GCGGTGGCACCTTGGTAG-3′) and G3PD_4307RC (5′-CCGTCCTTGTCCCGTTTCT-3′) and IDH_707F (5′-GGTACTTCGTGACCCTCCTGC-3′) and IDH_1436RC (5′-CGACCATAGTTGCGACCAGAC-3′) were designed for genes encoding Calreticulin (CRT, 808 bp), Glycerol-3-phosphate dehydrogenase (G3PD, 733 bp) and Isocitrate dehydrogenase (IDH, 729 bp), respectively. These three nuclear genes were amplified at 95 C 15 sec, 57 C 15 sec and 72 C 60 sec for 40 cycles. The PCR products were treated using Exosap-IT then sequenced with an ABI 3130 sequencer following the manufacturer protocols.

### Borrelia burgdorferi real-time PCR Detection

Taqman real-time PCR assays were performed in a duplex format (to detect DNA of tick and *B. burgdorferi*) with a reaction volume of 20 μl, by using the Brilliant II QPCR Master Mix in a Stratagene MX3000P QPCR System (Agilent, La Jolla, Calif.). The detection of tick DNA served as an internal control with 300 nM of primers Tick16S_F (5′-AATACTCTAGGGATAACAGCGTAATAATTTT -3′) and Tick16S_R (5′-CGGTCTGAACTCAGATCAAGTAGGA-3′), and 125 nM of Tick16S_Probe (FAM-5′- AAATAGTTTGCGACCTCGATGTTGGATTAGGAT 3'- BHQ1). The detection of *B. burgdorferi* was modified from (Courtney *et al*. 2004) with 700 nM primers Borrelia23S_F (5′-CGAGTCTTAAAAGGGCGATTTAGT-3′) and Borrelia23S_R (5′-GCTTCAGCCTGGCCATAAATAG-3′), and 300 nM of Borrelia_Probe (HEX-5′- AGATGTGGTAGACCCGAAGCCGAGTG-3′-BHQ1). Cycling conditions included an initial activation of the Taq DNA polymerase at 95 °C for 10 min, followed by 40 cycles of a 15 s denaturation at 95 °C and a 1 min annealing-extension step at 60 °C.

### Phylogeny and population structure

DNA sequences were aligned using the ClustalW software. Individual mitochondrial haplotypes were identified and converted to Arlequin format using FaBox^43^. Phylogenetic analyses were conducted using maximum likelihood (ML) method in PAUP 4.0b10^44^.

Nuclear haplotypes for heterozygotes were reconstructed using PHASE 2.1^45^ with default parameters. Genetic structure was examined by an analysis of molecular variance (AMOVA), implemented in Arlequin version 3.1^46^. Structure analysis was performed using Structure^47^ and BAPS version 5.3^48^. Structure analysis was carried out with 10 independent runs per each K value (K = 1–17), with a 100,000 burn-in period and 900,000 Markov chain Monte Carlo iterations. These analyses were performed using a model with admixture, correlated allele frequencies and with no a priori information on the sample location of individuals. The *Δ*K approach was used to estimate the most likely K value. For BAPS, a spatial clustering algorithm and a mixture analysis of individuals without geographic information was chosen, with 10 replicates from K = 2 to K = 17 ran. When we run clustering of 17 sample populations, K = 4 was calculated based on nuclear genes and K = 2 was justified based on mtDNA. We, therefore, set the maximum number of clusters to 2, 3 and 4 for BAPS.

### Locality and climate data used for ecological niche modeling

We composed a data set of 1423 county-level reported occurrences of blacklegged ticks (See Supplementary S3)^49,50^. These occurrences were separated into two partitions based on genetic results: northern *I. scapularis* population (n = 825) and southern *I. scapularis* population (n = 598). For climate layers we used bioclimatic variables for the present, the Mid-Holocene (~6Ka) and the Last Glacial Maximum (~21Ka) available from the WorldClim database 1.4 (http://www.worldclim.org) and resampled these to a 25 arcminute resolution (c. 50 × 50 km). We selected a subset of bioclimatic variables that show a Pearson’s correlation of r < 0.7, and are deemed biologically meaningful based on life history knowledge of the model system, in this case, seasonality: bio2 = mean diurnal temperature range (mean of the monthly difference between the minimum and maximum temperature), bio8 = mean temperature of the wettest quarter, bio9 = mean temperature of the driest quarter, bio16 = precipitation of the wettest quarter, and bio17 = precipitation of the driest quarter^51,52^ (Supplementary S2 Table 4).

### Ecological niche modeling

We created ecological niche models for northern and southern *I. scapularis* populations with Maxent 3.3.3k^53^. We restricted the feature type to hinge features as this facilitates extrapolation^54^. The area between 20 and 55 degrees latitude and -60 and -130 degrees longitude was chosen as environmental background. Ecological niche models were projected on the current and past climate layers. Statistical significance of ecological niche models was confirmed^55^ by testing their AUC values against a null model based on random localities created with ENMTools 1.3^56^.

## Supplementary information

Supplementary Table S3.

Supplementary Information.
