# Supplementary material for: Northern and southern blacklegged (deer) ticks are genetically distinct with different histories and Lyme spirochete infection rates
Source: Sci Rep. 2020 Jun 24;10:10289. doi: 10.1038/s41598-020-67259-0 (PMC7314838; doi:10.1038/s41598-020-67259-0)
Supplement: Supplementary file 2 — Supplementary Information. [file 41598_2020_67259_MOESM2_ESM.pdf]

Scientific Reports

Northern and southern blacklegged (deer) ticks are genetically distinct with different histories and Lyme spirochete infection rates

Guang Xu[1*], Ben Wielstra[2,3], Stephen M. Rich[1]

[1] Department of Microbiology, University of Massachusetts, Amherst, United States of America

[2] Institute of Biology Leiden, Leiden University, Leiden, The Netherlands

[3] Naturalis Biodiversity Center, Leiden, The Netherlands

* Corresponding author

E-mail: gxu@umass.edu (GX)

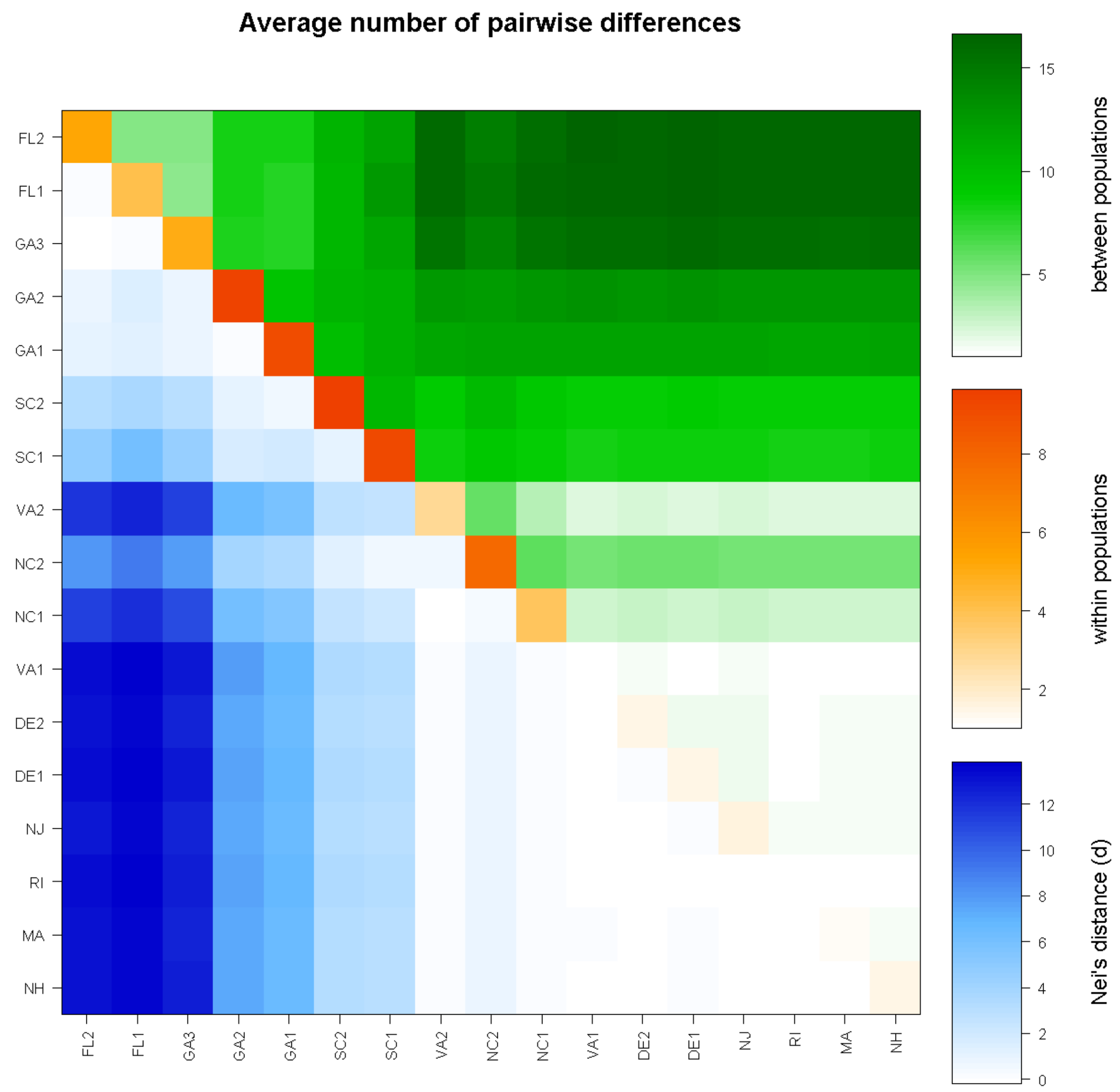

Supplementary S1 Fig A. Pairwise comparisons of 17 populations using mtDNA 16S gene.

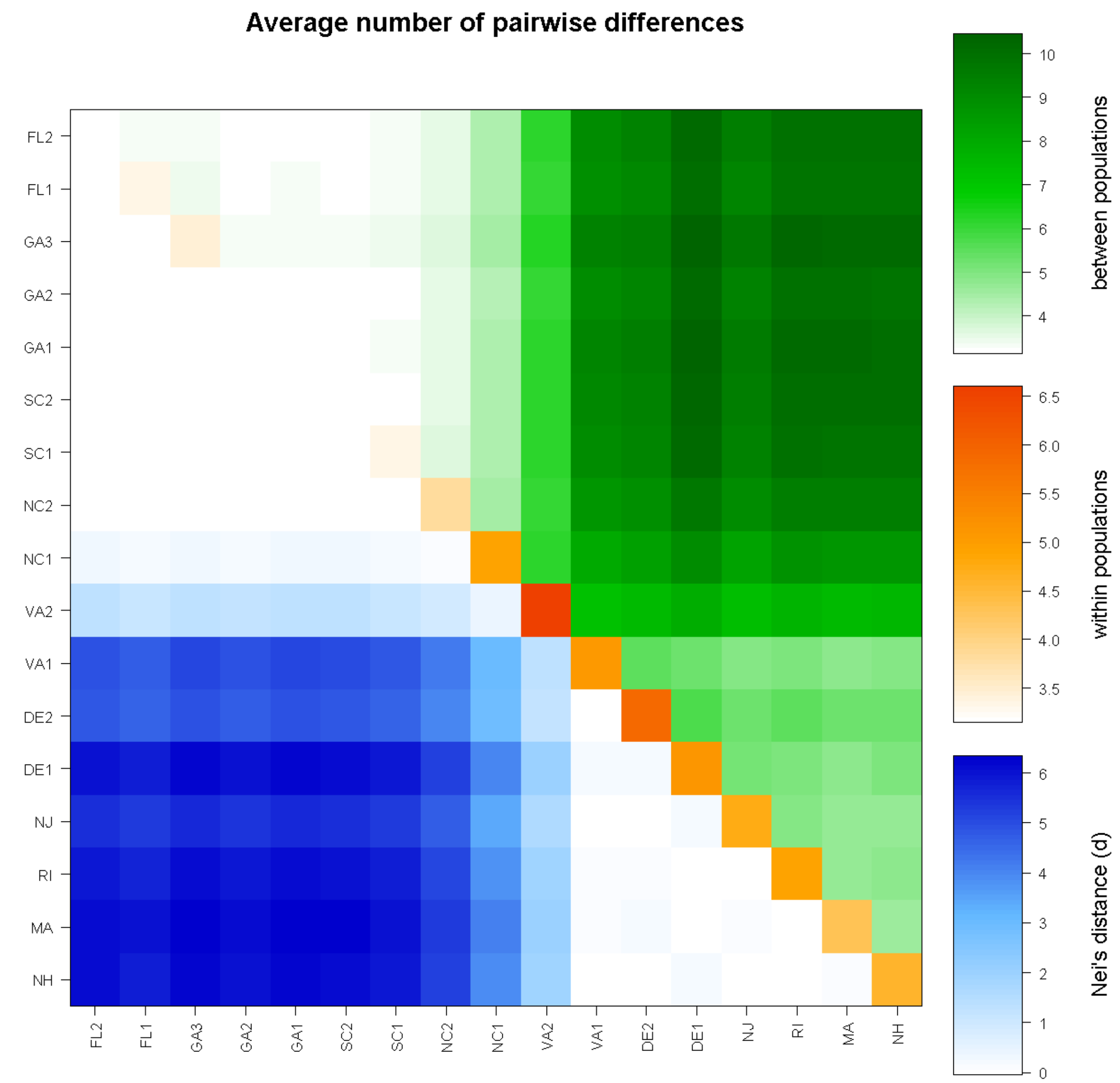

Supplementary S1 Fig B. Pairwise comparisons of 17 populations using nuclear CRT gene.

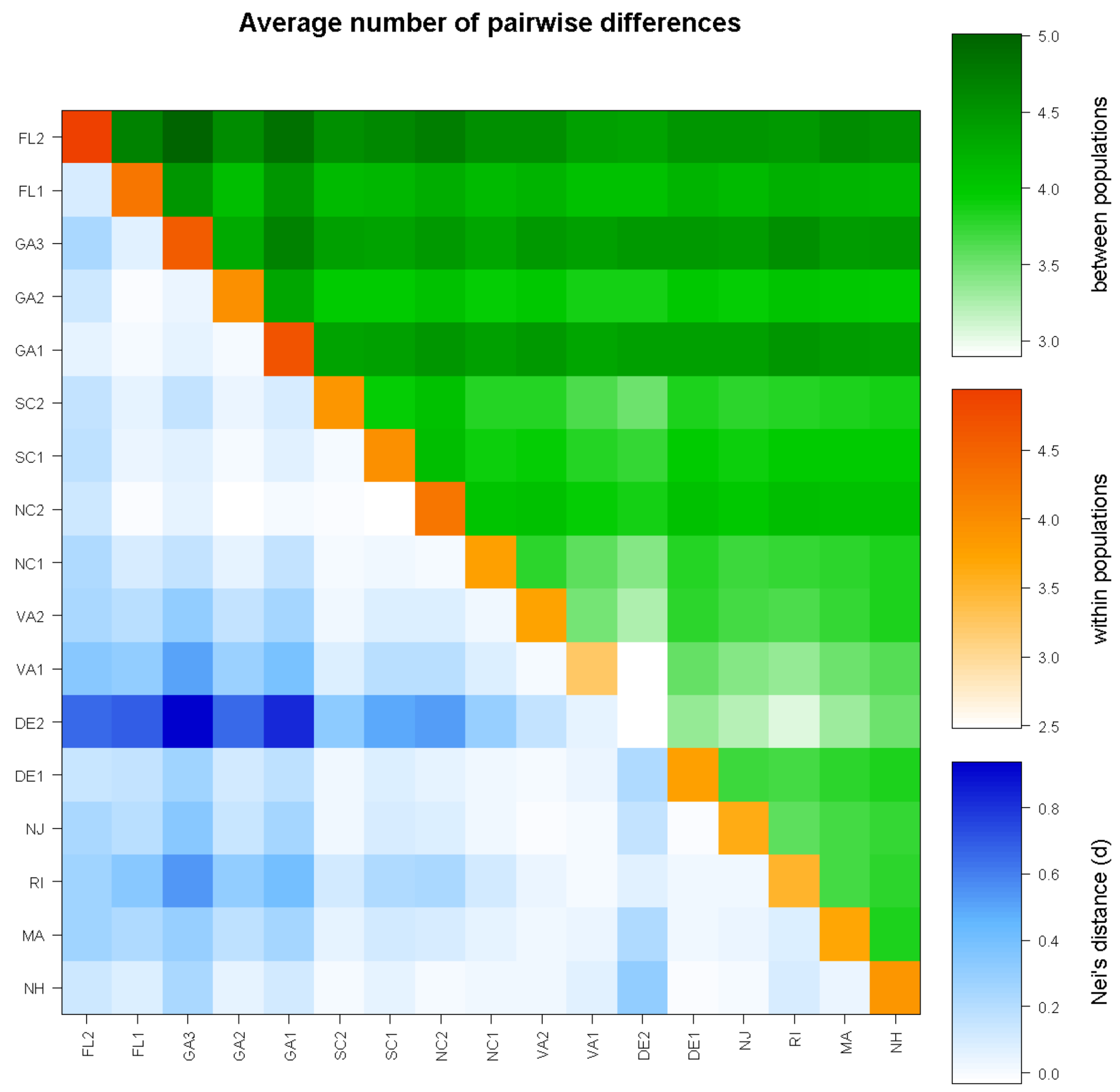

Supplementary S1 Fig C. Pairwise comparisons of 17 populations using nuclear G3PD gene.

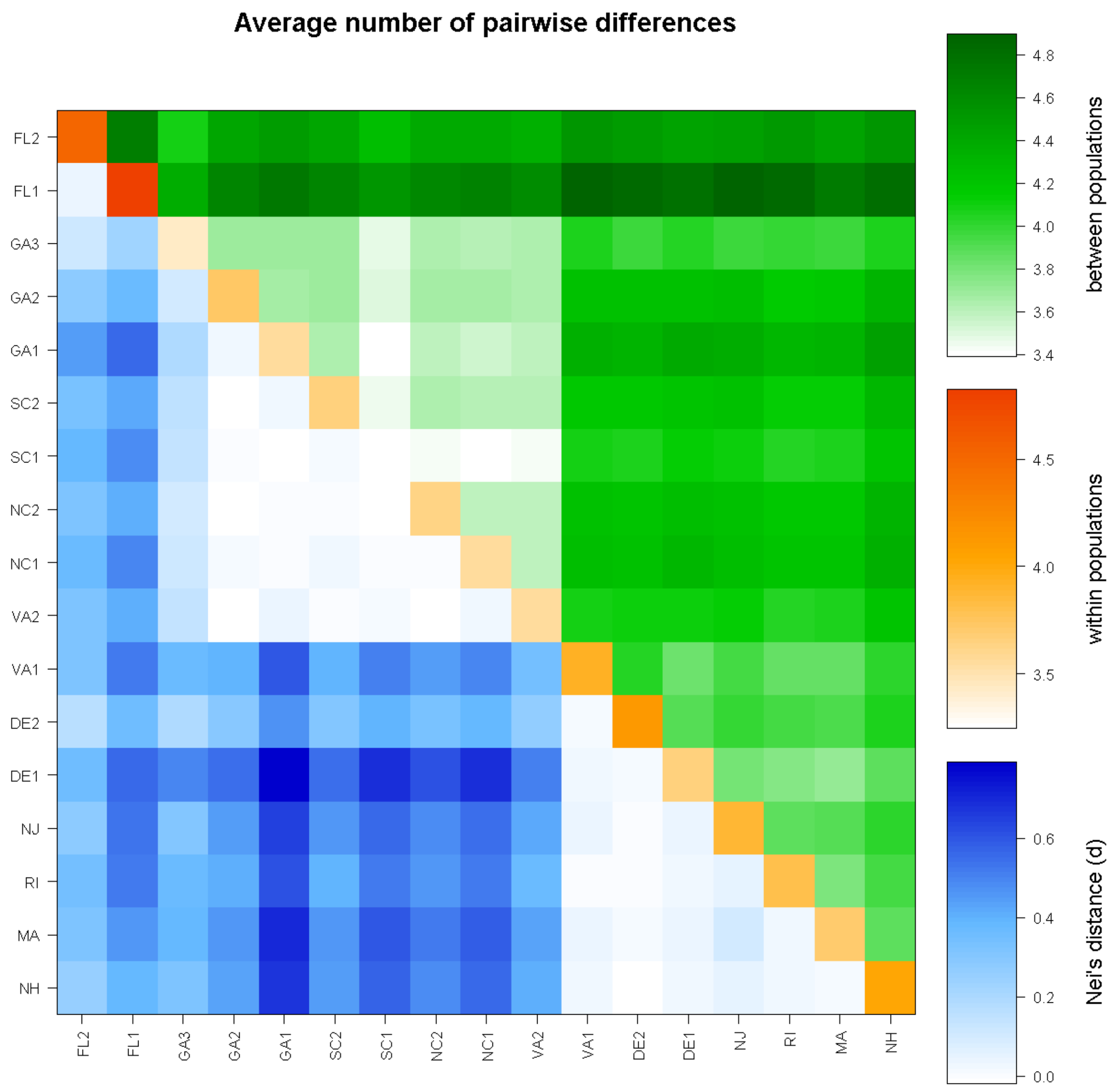

Supplementary S1 Fig D. Pairwise comparisons of 17 populations using nuclear IDH gene.

Supplementary 2 Table 1. Molecular diversity indices within the mt 16S rRNA gene for 17 *Ixodes scapularis* populations.

Population	Theta(Hom)	Theta(K)	Theta(S)	Theta(Pi)
FL1	3.617109	9.184249	6.472691	4.058385
FL2	4.111811	5.171673	5.76075	5.238095
GA3	3.467574	6.068464	6.008398	4.97076
GA2	4.082188	8.671562	5.638116	9.504386
GA1	3.097036	4.211906	5.433937	9.046253
SC2	3.650125	11.499964	6.839788	9.643048
SC1	3.361519	11.277806	5.746004	9.19332
NC2	10.150861	14.660475	5.757215	7.819355
NC1	7.372952	11.987522	5.437542	3.802469
VA2	4.678189	9.107343	5.794556	2.866059
VA1	1.665445	2.540178	1.365204	1.000000
DE2	2.239167	3.041918	1.625658	1.509881
DE1	2.720205	3.467044	1.676824	1.429429
NJ	4.286126	6.289422	2.923151	1.546971
RI	1.359705	2.735013	1.396098	1.016246
MA	1.469944	2.333035	1.591806	1.207387
NH	3.467415	6.653496	2.387709	1.42052

Supplementary 2 Table 2. Analysis of molecular variance comparing between and within north and south population of *Ixodes scapularis*.

Source of variation	df	Sum of Squares	F(prob)	Variance	%
CRT					
Between North and South	1	29.503	0.04898***	0.02661	5.33
Among collections within North and South	15	19.364	0.01124***	0.00600	1.20
Within collection	2427	1131.604	0.05967***	0.46626	93.46
G3PD					
Between North and South	1	8.278	0.01406***	0.00574	1.41
Among collections within North and South	15	15.521	0.01406***	0.00473	1.16
Within collection	2407	958.342	0.02562***	0.39815	97.44
IDH					
Between North and South	1	17.334	0.02750***	0.01253	2.57
Among collections within North and South	15	22.974	0.01647***	0.00782	1.60
Within collection	2435	1137.459	0.04175***	0.46713	95.83

16S					
Between North and South	1	16.297	0.05315***	0.02397	5.31
Among collections within North and South	15	34.774	0.06491***	0.02771	6.15
Within collection	1215	485.076	0.11461***	0.39924	88.54

*** P<0.0001

Supplementary 2 Table 3. Demographic Parameters based on 16S, CRT, G3PD and IDH genes.

	16S			CRT			G3PD			IDH		
	Tau	Theta	SSD	Tau	Theta	SSD	Tau	Theta	SSD	Tau	Theta	SSD
FL2	2.890	0.019	0.025	0.305	0.00	0.003	5.744	0.004	0.048	5.880	0.000	0.003
FL1	0.000	0.000	0.806	1.474	0.868	0.006	6.836	0.000	0.031	5.955	0.002	0.008
GA3	0.000	0.000	0.800	2.594	0.986	0.000	5.961	0.004	0.026	2.770	0.000	0.011
GA2	18.537	0.002	0.063	1.824	0.000	0.036	5.598	0.000	0.038	2.441	1.380	0.003
GA1	18.758	0.000	0.096	2.979	0.000	0.000	5.607	0.000	0.021	1.722	0.000	0.063
SC2	18.633	0.002	0.102	2.313	0.766	0.000	5.938	0.005	0.056	2.561	1.385	0.000
SC1	17.711	0.002	0.078	1.709	0.000	0.061	5.855	0.000	0.051	2.184	1.266	0.001
NC2	1.869	0.000	0.059	1.039	0.000	0.145	5.555	0.000	0.050	2.609	1.534	0.006
NC1	1.400	0.869	0.004	1.335	0.000	0.147	6.211	0.000	0.040	3.313	0.445	0.001
VA2	2.016	0.000	0.002	7.168	1.276	0.003	6.406	0.000	0.040	2.871	0.863	0.000
VA1	1.063	0.025	0.006	4.414	2.257	0.006	6.678	0.000	0.041	4.203	0.193	0.002
DE2	1.969	0.004	0.023	7.547	0.139	0.005	0.000	0.000	0.394	4.406	0.002	0.007
DE1	1.344	0.000	0.006	7.133	0.000	0.003	6.125	0.000	0.029	4.559	0.000	0.004
NJ	1.705	0.000	0.006	4.969	1.218	0.005	6.184	0.000	0.034	4.508	0.000	0.004
RI	1.064	0.000	0.002	6.568	0.007	0.006	7.396	0.002	0.060	3.973	0.000	0.003
MA	1.787	0.000	0.014	5.969	0.000	0.011	6.094	0.000	0.057	3.947	0.005	0.001

NH	1.549	0.000	0.005	5.640	0.638	0.008	5.844	0.000	0.034	4.355	0.007	0.004

Parameters tau is time since expansion expressed in units of mutational time; theta is time before and after population expansion; SSD means Sum of Squared deviation.

Supplementary 2 Table 4. Variable importance for the Ecological niche model

	South	South	North	North	North+South (All)	North+South (All)
Variable	Percent contribution	Permutation importance	Percent contribution	Permutation importance	Percent contribution	Permutation importance
bio_2	4.8	3.5	5.5	16.8	10.6	14.7
bio_8	7.1	3.1	20.4	15.3	10.5	10
bio_9	37.2	71.1	17.8	22.9	5.5	21.3
bio_17	0.9	1.8	41.6	41.4	47.3	16.1
bio_16	50.1	20.6	14.7	3.6	26.1	38

BIO2 = Mean Diurnal Range (Mean of monthly (max temp - min temp))

BIO8 = Mean Temperature of Wettest Quarter

BIO9 = Mean Temperature of Driest Quarter

BIO16 = Precipitation of Wettest Quarter

BIO17 = Precipitation of Driest Quarter
